# Dynamic representation of multidimensional object properties in the human brain

**DOI:** 10.1101/2023.09.08.556679

**Published:** 2024-06-13

**Authors:** Lina Teichmann, Martin N. Hebart, Chris I. Baker

**Affiliations:** 1 Laboratory of Brain and Cognition, National Institute of Mental Health, National Institutes of Health, Bethesda MD, USA; 2 Vision and Computational Cognition Group, Max Planck Institute for Human Cognitive and Brain Sciences, Leipzig, Germany; 3 Department of Medicine, Justus Liebig University Giessen, Giessen, Germany; 4 Center for Mind, Brain and Behavior (CMBB), Universities of Marburg, Giessen, and Darmstadt, Germany

## Abstract

Our visual world consists of an immense number of unique objects and yet, we are easily able to identify, distinguish, interact, and reason about the things we see within a few hundred milliseconds. This requires that we integrate and focus on a wide array of object properties to support specific behavioral goals. In the current study, we examined how these rich object representations unfold in the human brain by modelling time-resolved MEG signals evoked by viewing single presentations of tens of thousands of object images. Based on millions of behavioral judgments, the object space can be captured in 66 dimensions that we use to guide our understanding of the neural representation of this space. We find that all dimensions are reflected in the time course of response with distinct temporal profiles for different object dimensions. These profiles fell into two broad types, with either a distinct and early peak (~125 ms) or a slow rise to a late peak (~300 ms). Further, early effects were stable across participants, in contrast to later effects which showed more variability, suggesting that early peaks may carry stimulus-specific and later peaks more participant-specific information. Dimensions with early peaks appeared to be primarily visual dimensions and those with later peaks more conceptual, suggesting that conceptual representations are more variable across people. Together, these data provide a comprehensive account of how behaviorally-relevant object properties unfold in the human brain and contribute to the rich nature of object vision.

## Introduction

A central aspect of vision is our ability to identify, distinguish, interact, and reason about a huge variety of different objects. What neural representations support such a challenging task in the span of just a few hundred milliseconds? While many studies have focused on broad distinctions between objects (i.e., categories), it is clear that our understanding of objects extends well beyond our ability to label or discriminate them. Here, we used the publicly available THINGS dataset (Hebart, Contier, Teichmann et al., 2023) to determine the temporal dynamics of object vision in the human brain. We combined the large-scale THINGS-MEG dataset with a multidimensional model of object processing derived from millions of behavioral responses to gain a comprehensive understanding of the temporal unfolding of object vision.

To understand the temporal dynamics of object vision, prior studies have typically compared responses to different types of object stimuli (e.g., faces, scenes). For example, EEG and MEG studies have revealed differences in responses to faces and other objects that peak around 170 ms (Liu et al., 2002; Rossion, 2014). More recently, using multivariate and machine learning analyses, studies have further shown that M/EEG signals evoked by broad object categories (e.g., animals, plants, body parts) can be distinguished within the first 200 ms, focusing on what might be driving differences in the neural response (Carlson et al., 2013; Cichy et al., 2014; Clarke et al., 2013; Goddard et al., 2016; Grootswagers et al., 2019; Hebart et al., 2018; van de Nieuwenhuijzen et al., 2013). For example, to disentangle which object features contribute to differences in the neural signal, some studies have used stimulus sets with perceptually similar stimuli (e.g., glove and hand) or stimuli that straddle category bounds of object properties such as animacy (e.g., robots) (Contini et al., 2021; Kaiser et al., 2016; Proklova et al., 2019). Others have tried to separate the contribution of visual and semantic object properties to the neural signal by using cross-exemplar generalization to determine when we can distinguish objects across different exemplars (Bankson, Hebart et al., 2018; Carlson et al., 2013), across object position and size (Isik et al., 2014), or modelling the data using visual and semantic models (Clarke et al., 2015). Overall, the results of these studies suggest that early responses (<150 ms) reflect primarily visual feature information, with later responses reflecting more conceptual or semantic information.

While these studies have revealed some general features of the object response, they are often based on relatively small, hand-selected sets of stimuli which do not sample the object space in a representative way and cannot adequately capture the richness of the object response (Grootswagers & Robinson, 2021). Hand-selecting sets of stimuli may lead to a sampling bias because some types of objects are considered to be important (e.g., faces, animals) and may be overrepresented while others may be completely absent or underrepresented (e.g. furniture, cars, birds). To provide a more comprehensive understanding of object vision, we focused on addressing two key challenges: 1) sampling an expansive set of object stimuli across the thousands of object types we can identify and interact with, and 2) accounting for the rich meaning and behavioral relevance associated with individual objects beyond discrete labels. To do this, we turned to THINGS-data (Hebart, Contier, Teichmann et al., 2023), which contains MEG data for 1,854 objectively-sampled object concepts (Hebart et al., 2019) as well as rich behavioral data comprising 4.7 million similarity judgments that have been used to derive 66 underlying dimensions of objects in a data-driven manner (e.g., colorful, plant-related, transportation-related) (Hebart et al., 2020, Hebart, Contier, Teichmann et al., 2023). These dimensions reflect behavior-derived core features of the mental representations of these objects and incorporate both visual and semantic properties. Previous time-resolved analyses of response to the THINGS stimuli (THINGS-MEG: Hebart, Contier, Teichmann et al., 2023) and THINGS-EEG (Grootswagers et al., 2022) have revealed differential neural signals evident within the first 200 ms (Gifford et al., 2022; Grootswagers et al., 2022; Hebart, Contier, Teichmann et al., 2023) that enable object decoding. As with previous work, however, such analyses do not reveal what object properties drive these effects and the degree to which the relative contribution of different properties varies over time.

Here, we developed a novel approach to uncover the temporal dynamics of object processing by directly examining how behavior-derived object dimensions are reflected in the dynamic object representations in the human brain. These object dimensions are behaviorally-relevant, in that they support the key factors underlying arbitrary categorization behavior and as such underlie our ability to make sense of our visual world, to generalize, structure our environment, and to communicate our knowledge. We identified timecourses of neural information processing associated with each of 66 dimensions reported for THINGS (Hebart et al, 2020). In contrast to previous work requiring category-based stimulus selection and labelling, we use behavioral embeddings that characterize each image across multiple dimensions capturing similarity relationships directly. In addition, the dimension values for each object are continuous (e.g., jellybeans are more colorful than sunflowers, but sunflowers are more colorful than sugar), allowing for a fine-grained way of modelling similarity in the neural data and thus capturing the richness of object vision. In contrast to common approaches such as representational similarity analysis (RSA), our method allows us to directly study evoked neural representations at the global level (i.e., across all dimensions) as well as at the local level (i.e., each dimension separately). Critically, our approach goes beyond studying object identification and categorization and allows us to determine the time course of response specific to each of the behaviorally-relevant dimensions.

Our results show distinct temporal profiles for every single dimension. These temporal profiles tended to group according to the relative strength of two phases of processing (~125 ms and ~300 ms) as well as the presence or absence of an offset related response (~500–600 ms). Critically, early effects were more generalizable across participants while later effects were more variable across people. This suggests that stimulus-specific information is reflected in the early parts of the signal while subject-specific information unfolds later in time. An exception to this were dimensions that are primarily associated with physical properties of the object which generalized well across participants throughout the timeseries. Collectively, by focusing on behavioral relevance of object properties, our approach provides a comprehensive characterization of the temporal unfolding of visual object responses in the human brain.

## Results

The overarching goal of the current study was to characterize how multidimensional representations unfold over time by combining large-scale MEG data with behaviorally-relevant similarity embeddings. Our primary aims were to (1) extract timecourses from the MEG response that are associated with each behaviorally-relevant multidimensional profile, (2) reveal how these timecourses vary across dimensions and participants, and (3) identify prototypical temporal characteristics shared between response profiles of individual dimensions.

THINGS-MEG measured evoked neural responses in four participants viewing >27,000 unique natural images associated with 1,854 object concepts. To associate object dimensions with these natural images, we used behavioral embeddings derived from similarity judgments, based on 4.7 million judgments on 1,854 object concepts in a triplet odd-one out task (Hebart, Contier, Teichmann et al., 2023). Thus, the stimuli used in the MEG study are associated with both object concept labels (e.g., nail polish) as well as weights on behaviorally-relevant dimensions (e.g., colorful) (Hebart et al., 2020). The dimensions cover a broad range of object properties, with some being strongly linked to visual features (e.g., colorfulness) and others linked more to functional or contextual features (e.g., childhood-related). The behavioral similarity embeddings were based on concept-level judgments (i.e., one image per object concept), potentially missing some of the visual variability in the MEG stimuli. To overcome this issue, we used an artificial neural network (CLIP-VIT, Radford et al., 2021) to augment the behavioral dataset and generate image-level embeddings for later predictions (Hebart et al., 2022). Post-hoc analyses showed a consistent improvement in prediction scores for all 66 dimensions when using image-level versus concept-level embeddings. While the results were consistently stronger, the overall pattern of results remained similar even without the use of CLIP-VIT, specifically for more semantic dimensions.

We used the scores on the 66 behaviorally-relevant dimensions to model the evoked neural response to >26,000 images recorded with MEG. In particular, we used both decoding and encoding models to associate the multivariate MEG-sensor response with the 66-dimensional behavioral embedding. In contrast to previous work, our method allows us to effectively examine *multidimensional* object profiles that are obtained from behavioral data in a data-driven way. We can capture and examine the relationships between objects across many dimensions, as two images that are similar along one dimension may be very different along another dimension (e.g., beads and nail polish are both colorful but not necessarily childhood-related). Thus, this approach does not rely on selecting and contrasting object classes but instead uses the same images and experimental trials with a relabeling according to behaviorally-relevant dimensional profiles.

### (1) Distinct time courses can be derived for each behaviorally-relevant dimension

**Figure 1.**
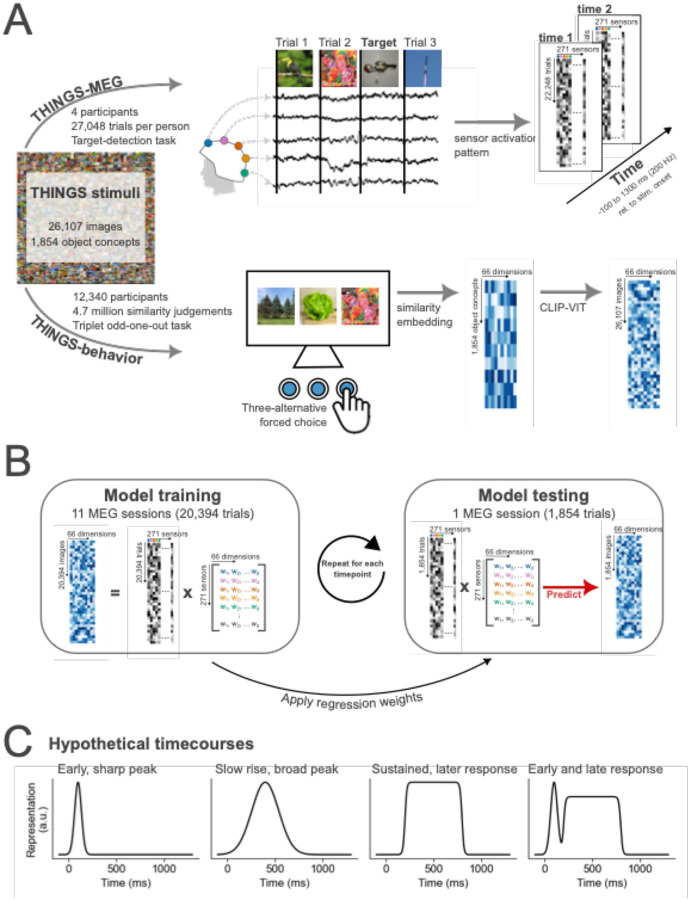
Summary of THINGS-MEG and THINGS-Behavior datasets and the methodological approach to combine them. (A) Summary of the datasets used. Evoked responses to images from the THINGS image-database were recorded over time using MEG. In total, four participants completed 12 sessions, resulting in >100,000 trials in total. During the MEG session, participants were asked to detect computer-generated images of non-nameable objects. In the behavioral task, a separate set of participants viewed three objects from the THINGS image-database at the time and were asked to pick the odd-one-out. A computational model was then trained to extract similarities along 66 dimensions for all object concepts. Using CLIP-VIT, we extended the embedding to capture similarities for every image. The data for behavioral data was crowdsourced via Amazon Mechanical Turk. In total >12,000 participants completed a total of 4.7 million similarity judgements. (B) Overview of the methodological approach of combining these two datasets with the goal of understanding how multidimensional object properties unfold in the human brain. To train the model, we extract the sensor activation pattern at each timepoint across the MEG sensors and use the behavioral embeddings to learn an association between the two datasets. The linear regression weights are then applied to sensor activation patterns of independent data to predict the behavioral embedding. To evaluate the model’s performance, we correlated the predicted and true embedding scores. (C) Hypothetical timecourses that could be observed for different dimensions.

**Figure 2.**
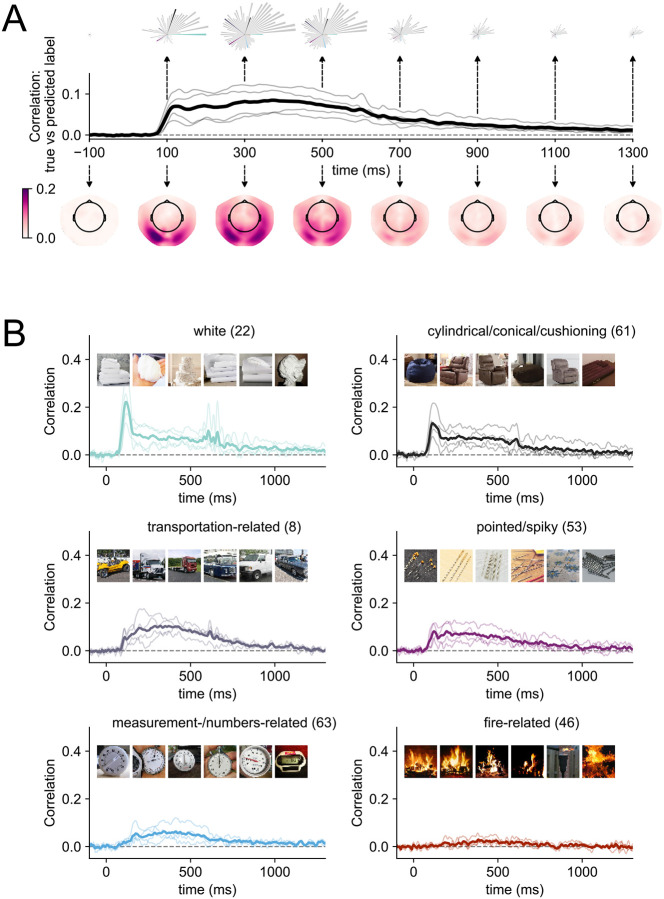
Modelling results for within-participant models of MEG data and multidimensional similarity judgments. (A) Correlation between the predicted and true behavioral embeddings across all dimensions over time. The thick, black line shows the average across all participants, the thin grey lines individual participants. The rose plots above the timeseries show snapshots of the model performance for each of the 66 dimensions at individual time points. The order of the petals is based on maximum peak amplitude across time (i.e., dimension “white” has the largest peak of all 66 dimensions). Longer petals indicate that there is more information associated with a given dimension in the signal at this timepoint. Petals that are highlighted with individual colors are presented in more detail in panel B. The topographical maps below the timecourses show temporal snapshots of the model performance when it is fitted to individual sensors. Darker colors show a higher correlation between the predicted and true weights at each sensor location. A dynamic version of the topographical plots can be accessed here. (B) Example timecourses for six dimensions. Timecourses were first sorted by peak amplitude, and then we picked every eleventh timecourse to show a representative sample of timecourses with different signal-to-noise ratios. The images within each subplot show the top six stimuli on that dimension. All individual timecourses can be found in the Supplementary Materials (Figure S1). A dynamic version of the rose plots can be accessed here.

We fitted multiple linear regression models to learn the association between the MEG sensor activation patterns and the behavioral embeddings at every timepoint (Figure 1). The linear models were fit on MEG data from 11 sessions (20,394 trials). Using the left-out, independent session (1,854 trials) as a test set, we predicted the continuous value along each dimension from the MEG sensor activation patterns. Correlating these predicted scores with the true behavioral dimensional profiles resulted in timeseries of dimension information in the neural response for all four participants. The analysis revealed behaviorally-relevant multidimensional information from ~80 ms onwards (Figure 2A). The timecourse showed an early peak at ~100 ms which was maintained over time up to 1000 ms after stimulus onset, with an overall peak at ~ 300ms. To examine the relevance of individual MEG sensors to this effect, we also fitted linear models to predict each sensor activity pattern separately. In particular, we trained models to predict the univariate response in each MEG sensor at every timepoint using the multidimensional behavioral values associated with each stimulus. Our results showed that, while posterior sensors had the strongest effect across time, the relative contribution of frontal sensors increased later in time (>150 ms).

Extracting the correlation timecourses for each dimension separately, our results revealed a different unfolding of neural responses across time for different dimensions (Figure 2A rose plots, Supplementary Figure S1). For example, dimensions such as “plant-related”, “colorful/playful”, “white” showed distinct, early peaks (~125 ms). In contrast, other dimensions such as “body-/people-related”, “food-related”, and “transportation-related” yielded a slower rise to a later peak (~300 ms). In addition, several of the dimensions yielding distinct early peaks exhibited a stimulus offset effect at ~500 ms. In contrast, several other dimensions did not show a distinct early peak or offset response but unfolded slowly over time and rose to a late peak (>300 ms). While signal-to-noise ratio differed across dimensions, all dimension timecourses exceed zero at some point over time (see Figure 2B for representative example timecourses selected based on peak amplitude and Supplementary Figure 1 for all timecourses). Strikingly, the behaviorally-relevant dimensional profiles were evident in the neural response even though MEG participants completed an orthogonal detection task. This demonstrates that the dimensions are automatically reflected in the neural data without a task that requires participants to engage with the object properties directly. Overall, these results highlight that a wide range of behaviorally-relevant dimensions are reflected in distinct temporal profiles and that their information is distributed across MEG sensors.

### (2) Timecourses vary across dimensions and are consistent across participants

**Figure 3.**
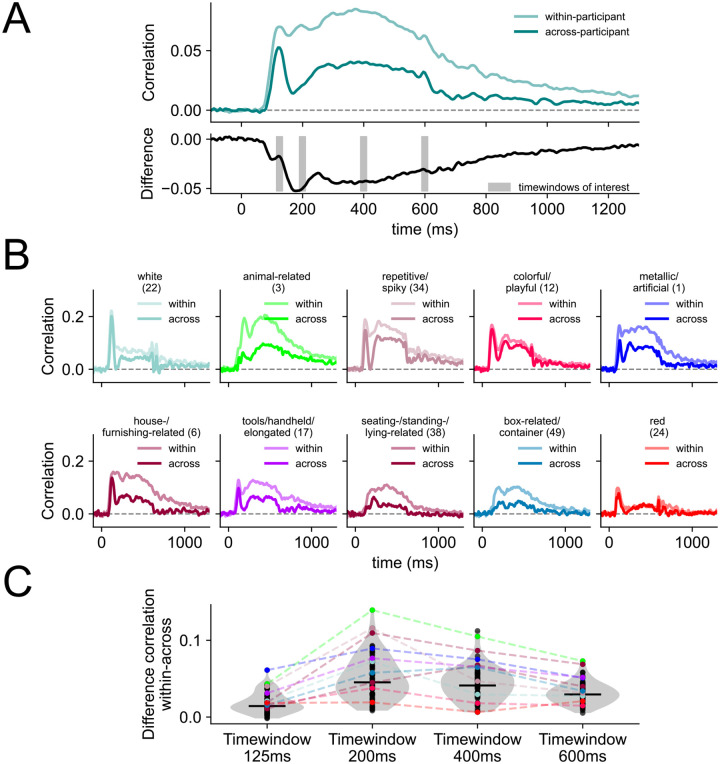
Differences between within- and across-participant model. (A) Average performance of the model across all dimensions when fitted as a within-participant model (session-wise cross-validation) and an across-participant model (participant-wise cross-validation). The black line below shows the difference between the two. Timewindows of interest around 125 ms, 200 ms, 400 ms, and 600 ms are highlighted which we used to examine the differences between the within- and across-participant model in more detail (see C). (B) Each subplot shows an example dimension timeseries when the model is fit within each participant (light color) and across different participants (dark color). (C) Comparison of the differences between the within- and the across-participant model at four timewindows of interest. We selected the first timewindow based on the peak within-participant correlation, and then additional timewindows at regular intervals. Every dot shows the difference between the within and across model for each of the 66 dimensions. The example dimensions from (B) are highlighted in their corresponding colors.

Building on the finding that a range of behaviorally-relevant dimensions are reflected in distinct neural profiles measured with MEG, we next tested to what degree these timecourses are consistent across participants. We used multiple linear regression models (see (1)), but this time with a subject-based cross-validation scheme, to examine whether the temporal characteristics we found for each dimension are idiosyncratic or consistent across participants. Specifically, we trained the model on MEG data from three participants and tested its performance on the data from the remaining one. This is a very stringent test for generalizability, as the model trains and tests on completely different datasets. Our results show that for most dimensions the across-participant models revealed similar timeseries characteristics as the within-participant model (Figure 3), highlighting that the temporal profiles we uncovered for each dimension were robust and not idiosyncratic to specific individuals of our study. As Figure 3A shows, the early peaks (~125 ms) in particular were consistent in amplitude and timing when comparing the within- and the across-participant model. In contrast, later effects (e.g., 200 ms, 400 ms, 600 ms) did not generalize as well across participants for most dimensions. For many dimensions, we observed a substantial drop in performance for the across-participant model performance relative to the within-participant model at around 200 ms before the performance improved again (Figure 3B & 3C). This indicates that the differences we observed between the within- and across model were not solely driven by a time-dependent decrease in signal-to-noise ratio. The magnitude of the differences between the within- and across-participant models were stable after the initial drop from around 250 ms onwards (Figure 3C). In addition, the results show that strong stimulus-offset effect at 500–600 ms observed in some dimensions for the within-participant models (e.g., the color dimensions) were also present when the model was trained and tested across participants. Together, these findings suggest that early effects (~125 ms) may carry largely stimulus-specific information that generalizes well across participants, while slightly later effects (~200 ms) are more subject-specific. However, for dimensions that are visually more homogenous (e.g., white, colorful), we found that the within- and across-models perform similarly throughout time, including the response associated with stimulus offset.

### (3) Peak timing and relative amplitude are prototypical temporal characteristics of different dimension timecourses

**Figure 4.**
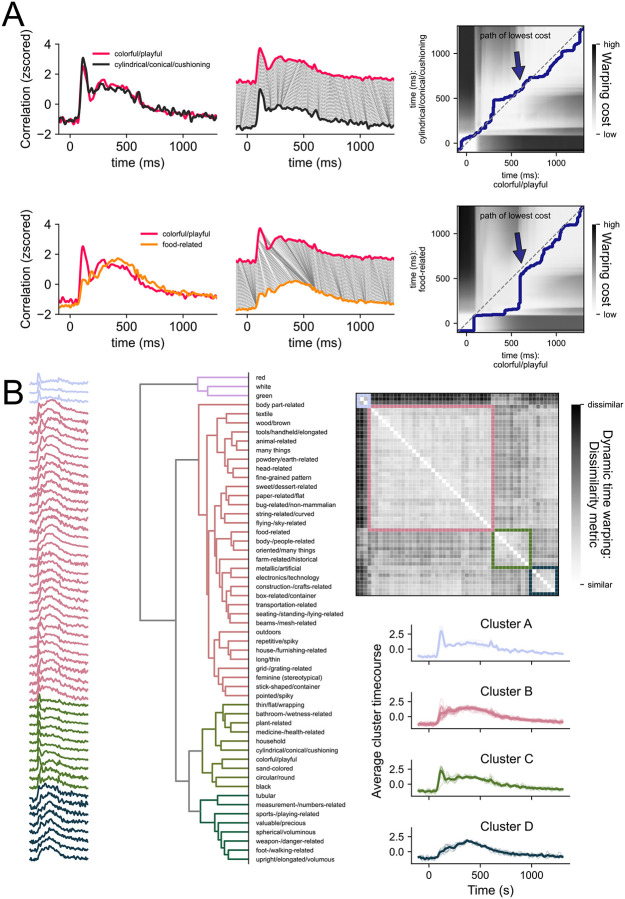
Dynamic time warping (DTW) as a method to compare timeseries similarities and extract prototypical timeseries characteristics. (A) shows the DTW approach for two pairs of timeseries. The correlations were scaled and plotted over time (left panel). DTW assesses how much one timeseries needs to be warped to resemble the other one (middle panel). This warping cost can be established by calculating the Euclidean distance between all timepoints (top right panel) to assess dissimilarity. The path of lowest cost describes the path through the matrix that minimizes the warping costs while adhering to some rules (see Methods). To summarize the warping cost in a single number, we summed the Euclidean distances along the path as a dissimilarity measure. (B) shows the dissimilarity matrix containing the DTW similarity measures for timeseries pairs (right panel). Timeseries with low signal-to-noise ratio were excluded (see methods). Hierarchical clustering on this matrix allows us to sort the dimension timeseries (left panel) and dimensions labels (middle panel). Averaging the timeseries for each cluster (bottom right panel) allows to examine prototypical timeseries characteristics.

Comparing the dimension timecourses visually suggests some commonalities across dimensions. For example, some dimensions shared a strong early peak and others showed a slower, gradual rise. To quantify the similarity of timecourse shapes across dimensions, we used dynamic-time warping (DTW, Chu et al., 2002). DTW captures the similarity between a pair of timeseries by assessing how much one of the timeseries has to be warped to resemble another one (Figure 4A). The result of this analysis is a time-time-matrix with cost values indicating the amount of warping that has to be done at every timepoint. To measure the similarity of a given timeseries pair, we extracted the sum of the Euclidean distances along the path of lowest cost. If the path falls on the diagonal of the time-time-matrix it means that the timeseries are identical. If it veers off the diagonal, the timeseries are more dissimilar (Figure 4A).

Applying DTW to our data, we generated a distance matrix for dimension timeseries pairs and ran hierarchical clustering on that matrix to determine which dimensions evoked similar time courses (Figure 4B). Cluster A first separated from all other dimensions. This cluster contained dimensions describing colors (“red”, “white”, “green”). Next, Cluster B separated from Cluster C and Cluster D. Cluster C contained dimensions describing colors (e.g., “sand-colored”, “black”) as well as other features such as shape (e.g., “circular/round”, “thin/flat”). After running the clustering, we sorted and averaged the cluster correlations to examine prototypical timeseries characteristics. The primary feature that appeared to distinguish the different time series was the relative strength of the early (~ 125 ms) and late (> 200 ms) correlations. The presence of an early peak (clusters a and c) was also often accompanied by a second local peak around the time of stimulus offset. Dimensions within these two clusters included “red”, “green”, “thin/flat”, and “colorful/playful”. These were all dimensions that appear to reflect a specific visual feature (e.g. color, shape) contained within the images. Thus, the early peak and strong offset response might be driven by underlying visual consistencies in objects with high scores on these dimensions.

In contrast, the other clusters showed the strongest correlation after 200 ms, with a slow rise to a late and prolonged maximum, but differing in the relative size of the early and late correlations. Dimensions within these clusters included “farm-related”, “flying-related”, and “body-/people-related”. Notably, these clusters accounted for the majority of the dimensions, highlighting the importance of the later response for behaviorally relevant features.

Overall, the clustering suggests that there are two broad classes of temporal profiles, those with a distinct, early peak and a stimulus offset effect and those with a late peak, often without any clear early peak. The clusters with strong early effects, which showed better generalization across participants, tended to reflect more stimulus-specific information (e.g. green, colorful). In contrast, the clusters with strong late peaks, which showed weaker generalization, appeared to correspond to more conceptual properties, possibly reflecting a greater contribution of subject-specific information.

## Discussion

Resolving incoming visual information to make sense of our environment is a challenging task that our brain solves within just a few hundred milliseconds. Here, we used a similarity embedding based on millions of behavioral judgements to model temporally-resolved neural responses in order to understand how the behaviorally-relevant features of objects are represented in the brain over time. Using THINGS-MEG (Hebart, Contier, Teichmann et al., 2023), we found that individual object dimensions were directly reflected in the neural response, with distinct temporal profiles for different dimensions. In particular, while some dimensions showed strong representation shortly after stimulus onset resulting in a pronounced early peak in the timeseries, other dimensions showed a slower rise to a later peak. For most dimensions, these early effects were highly similar across individuals allowing for across-participant generalization, while later effects were less consistent across participants. Some dimensions that are visually more homogenous (e.g., “red”) exhibited high across-participant generalization throughout time. Collectively, these results indicate that dimensions capturing physical stimulus properties evoke a more similar response across participants. Overall, our work highlights that behaviorally-relevant object properties emerge and evolve at different timepoints in the neural signal and contribute to the rich nature of object vision.

Our data-driven approach focused on behaviorally-relevant object dimensions contrasts with more typical category or feature-driven approaches. First, prior work has often used small, hand-selected stimulus sets (e.g., Bankson, Hebart et al., 2018; Carlson et al., 2013; Cichy et al., 2014; Grootswagers et al., 2018; Kriegeskorte et al., 2008; Teichmann et al., 2020) with findings that may be tied closely to the specific stimuli chosen (Grootswagers & Robinson, 2021). While larger stimulus sets potentially avoid this problem, it is often unclear how the stimuli were sampled, and biases within the stimulus set may still constrain the results. For example, the COCO stimuli used in the large-scale MRI Natural Scenes Dataset (Allen et al., 2022) heavily oversample 80 individual categories, leading to countless images of giraffes or surfers, which may also overestimate generalization performance (Shirakawa et al., 2024). Second, analyses in prior work often focused on category or feature labels assigned to individual stimuli, which ignore our broader understanding of objects and the specific properties that may be shared between different objects. One alternative approach used in prior work is to model the neural data using feature norms (e.g., McRae et al., 2005). In one particular MEG study (Clarke et al., 2015), this approach was used to model semantic content of stimuli from 11 categories which was then contrasted with output from a computational model of object vision. A drawback of this approach is that feature norms rely on verbally naming properties which means key visual or conceptual features may be missed while other features may be overemphasized. In contrast, our approach extracts object properties that are behaviorally-relevant using a visual odd-one-out task which does not require properties to be nameable and automatically extracts their individual relevance.

Our approach used the >27,000 images of the THINGS database which come with a behavioral embedding derived from 4.7 million similarity judgements. Using these datasets means reduced bias in stimulus selection and category assignment, as the THINGS concepts have been selected systematically (Hebart et al., 2019), and the behavioral embeddings were derived in a data-driven fashion, relying on crowdsourced similarity judgements (Hebart et al., 2020, Hebart, Contier, Teichmann et al., 2023). Instead of analyzing image-specific effects that reflect one category or another, we modeled the neural data for all object images in terms of continuous similarity scores along 66 dimensions. That means the MEG data going into the analysis always remained the same, but the label value capturing how strongly each image is associated with the dimension at hand differed. This approach is powerful as it makes use of all the data while allowing to study many dimensions simultaneously. Furthermore, it captures the complexities of object vision where objects are associated with many properties. The data here show that information related to all 66 behaviorally-relevant dimensions can be read-out from the MEG signal and have distinct temporal profiles. Together, our results highlight that modelling neural responses using continuous behaviorally-relevant embeddings offers a more comprehensive understanding of the visual object space than a focus on object category or individual, experimenter-selected dimensions.

We found different temporal dynamics for different object dimensions of visual object processing with a data-driven approach that broadly revealed two distinct temporal characteristics. We observe that some dimensions had a transient and distinct, early peak <150 ms and an offset response at ~100 ms after stimulus-offset. In contrast, other dimensions lacked the early peak completely or showed it more subtly. These dimensions tended to slowly rise to a later peak (~300 ms) that is more sustained over time. We found that early effects were more consistent across participants than later ones, suggesting that early peaks reflect stimulus-specific and later peaks subject-specific information. Indeed, when looking at which dimensions had a distinct early peak, we found that stimulus-specific visual properties such as color drove early distinctions. In contrast, later effects seemed to be more associated with concept-related properties, and critically our results suggest that the impact of such properties was variable across participants. It is important to note that the stimulus-specific effects we observed here are not tied to specific exemplars, as every unique image was shown only once and all analyses were based on cross-exemplar generalizations. Previous work has used cross-exemplar generalization as a method to disentangle visual and conceptual object properties (e.g., Bankson, Hebart et al., 2018; Carlson et al., 2013), however, this approach does not allow us to distinguish which object properties drive the effects at different timepoints. Our approach uses multidimensional behavioral embeddings and can therefore tease these differences apart by showing which behaviorally-relevant property contributes to the neural signal at a given timepoint. Overall, the results highlight that distinct temporal profiles are associated with different behaviorally-relevant dimensions but that some broad characteristics can distinguish between stimulus- and subject-specific information.

One limitation of our work is that the behavioral embedding was derived from a separate set of participants than those from which neural responses were collected. While our data is consistent enough to be generalizable across participants, we find that generalization performance is better for earlier peaks and dimensions that capture perceptually homogenous features (e.g., red, green, colorful). This may partially be the case because our behavioral embeddings are derived from crowdsourced data and thus may prioritize dimensions that tend to be shared across individuals. Future work should investigate individual differences more closely to understand how the object space may be skewed given personal experience and the task at hand.

In conclusion, by using behavioral judgments of similarity to guide our understanding of the neural representation of the object space, we find that different aspects of the object response emerge at different timepoints and together create the experience of meaningful visual object processing.

## Methods

### Dataset

We used the publicly available THINGS dataset (Hebart, Contier, Teichmann, et al., 2022) which contains densely sampled MEG data as well as crowdsourced behavioral data. The MEG portion of the data contained neural recordings from four participants who each viewed a total of 27,048 unique images over the course of a 12-sessions. Every image was shown centrally for 500 ms with an inter-stimulus interval of 800–1200 ms. Participants completed a target-detection task, looking for artificially generated images of objects that do not exist. Of the 27,048 trials, 22,248 trials were experimental trials showing unique images from the THINGS database (Hebart et al., 2019), which were used for the analysis.

Each image belonged to one of 1,854 object concepts (e.g., aardvark, clock, chicken wire, among many others). Unique image exemplars for each of the concepts were repeated 12 times over the course of the MEG experiment (one image per concept per session). In addition to the image concepts, we used a behaviorally-relevant embeddings to model MEG sensor responses. The embeddings contained weights on 66 dimensions which capture trial-by-trial responses for 4.7 million odd-one-out judgments on triplets of the 1,854 object concepts (Hebart, Contier, Teichmann et al., 2023). Each dimension describes a certain object property (e.g., circular/round, colorful, food-related), however, these dimensions were derived in a data-driven way based on the behavioral data. The original embedding was trained at the concept-level (one image per concept) and hence could miss visual variability across exemplars. In order to obtain image-level embeddings, we used a neural network model (CLIP-ViT, Radford et al., 2021) that can predict image-text pairs and has also been shown to be able to predict similarity judgments with high accuracy (Hebart et al., 2022; Muttenthaler et al., 2022). We started by examining the activity patterns in the final layer of the image encoder for each of the 1,854 objects. We then used ridge regression to predict dimension weights for each of the 66 dimensions for all images in the THINGS database. To model the evoked neural response measured with MEG, we then used the image-level predicted weights along the 66 dimensions. Please note that, while this analysis relies on features derived from a neural network model, the human similarity embedding showed good fits at the level of individual dimensions, demonstrating that these effects were not merely driven by projecting the CLIP image embedding to 66 arbitrary unidimensional spaces.

### Preprocessing

Our preprocessing pipeline was built using *mne-python* (Gramfort et al., 2013) and described in detail in the dataset release (Hebart, Contier, Teichmann, et al., 2022). The preprocessing steps included filtering (1Hz – 40Hz), baseline correction using z-scoring and epoching the data from −100 to 1300 ms relative to stimulus onset. Preprocessed data can be directly downloaded from OpenNeuro (https://openneuro.org/datasets/ds004212/versions/2.0.0).

### Analyses

#### Modelling MEG data based on multidimensional similarity judgments: within-participant regression

To model how the behaviorally-relevant dimensions unfold over time in the human brain, we fitted a multiple linear regression model at every timepoint to learn the association between the multivariate MEG-sensor response and the scores along each dimension. We trained the model on data from 11 out of the 12 sessions (20,394 trials) and tested on the remaining one (1,854 trials). This process was repeated so that every session was used as testing data once. The model was trained and tested for each participant separately. A separate model was trained and tested at every timepoint. Models were fit in Python using sci-kit learn linear regression models with default parameters (Pedregosa et al., 2011).

We assessed the model’s performance by correlating the predicted dimension score of all left-out trials with behavioral embeddings for each of the images. These correlations were interpreted as amount of information in the neural signal associated with a given dimension. We ran 10,000 iterations of the model fit with permuted weights in each dimension, to establish a 95% confidence interval representing chance model performance.

To gain insights into which sensors primarily drove the effects, we also trained a linear model to predict the activation of each sensor using the multidimensional similarity judgments. We used a session-wise cross-validation approach and ran this analysis for each participant separately. The model’s performance was assessed by correlating the predicted sensor activations for the test-set and the true sensor activations at every timepoint.

#### (b) Examining timecourse similarities across people: Across-participant regression

To examine whether timecourse profiles are consistent across participants, we also trained a model with a participant-wise cross validation scheme. We trained the model to learn the association between the multivariate MEG sensor activation pattern at every timepoint and the behavioral dimension profiles using data from three of the four participants. We then tested its performance using the data from the left-out participant. We repeated this process until every participant’s data were used as testing data once. A separate model was trained and tested at every timepoint. Models were fit in Python using *sci-kit learn* linear regression models with default parameters (Pedregosa et al., 2011).

#### (c) Examining timecourse similarities across dimensions: Dynamic Time Warping

The results of the regression models were timeseries of correlations for each dimension. To compare the shapes of these timeseries and assess overall similarities and differences, we used dynamic time warping implemented in the *dtaisdistance* toolbox (Meert et al., 2020). In contrast to correlation measures which are compression based, DTW is shape based and is well suited to investigate timeseries similarities that may have a temporal drift (Aghabozorgi et al., 2015). The goal of DTW is to find matches between patterns of two timeseries by assessing how much one timeseries has to be warped to look like the other one. This is achieved by generating distance matrices filled with pairwise Euclidean distances between timepoints and finding the shortest path through this matrix while adhering to several rules: The start and end of the timeseries have to align, the path cannot go back in time, and it has to be continuous.

The DTW similarity measure represents the sum of the Euclidean distances along the shortest path. We extracted this measure for smoothed timecourses and generated a similarity matrix. Given that we were interested in the relative shape of the timeseries and not the differences in signal-to-noise ratio, we normalized the timeseries before running the dynamic time warping by calculating z-scores for each dimension timeseries and each participant. Then we averaged across participants and calculated the DTW similarity measure for all dimension comparisons. Because z-scoring can amplify the effect of noisy timeseries, we sorted the timecourses by peak amplitude and excluded the bottom 12 timecourses from this analysis (see bottom two rows in Supplementary Figure S1). Running hierarchical clustering on the resulting DTW distance matrix allowed us to establish a qualitative measure of different prototypical timeseries characteristics. We set the threshold for the hierarchical clusters to be at 0.5 x the maximum distance observed.

### Open Science Practices

All data is publicly available under a Creative Commons license and can be downloaded from OpenNeuro: https://openneuro.org/datasets/ds004212/versions/2.0.0 *[this repository does not contain all aggregate behavioral data to run the analyses yet. It will be populated and re-uploaded as version 3.0.0 as soon as the review process is finalized]*. The analysis code for all analyses in this paper are available on GitHub: https://github.com/Section-on-Learning-and-Plasticity/THINGS-MEG.

## Supplementary Material

1
