# Supplementary material for: Dynamic representation of multidimensional object properties in the human brain
Source: bioRxiv. 2025 Feb 28:2023.09.08.556679. Preprint. [Version 4] doi: 10.1101/2023.09.08.556679 (PMC10515754; doi:10.1101/2023.09.08.556679)
Supplement: Supplement 1 [file NIHPP2023.09.08.556679v4-supplement-1.pdf]

bioRxiv preprint doi: https://doi.org/10.1101/2023.09.08.556679; this version posted February 28, 2025. The copyright holder for this preprint (which was not certified by peer review) is the author/funder. This article is a US Government work. It is not subject to copyright under 17 USC 105 and is also made available for use under a CC0 license.

Supplementary Materials

bioRxiv preprint doi: https://doi.org/10.1101/2023.09.08.556679; this version posted February 28, 2025. The copyright holder for this preprint (which was not certified by peer review) is the author/funder. This article is a US Government work. It is not subject to copyright under 17 USC 105 and is also made available for use under a CC0 license.

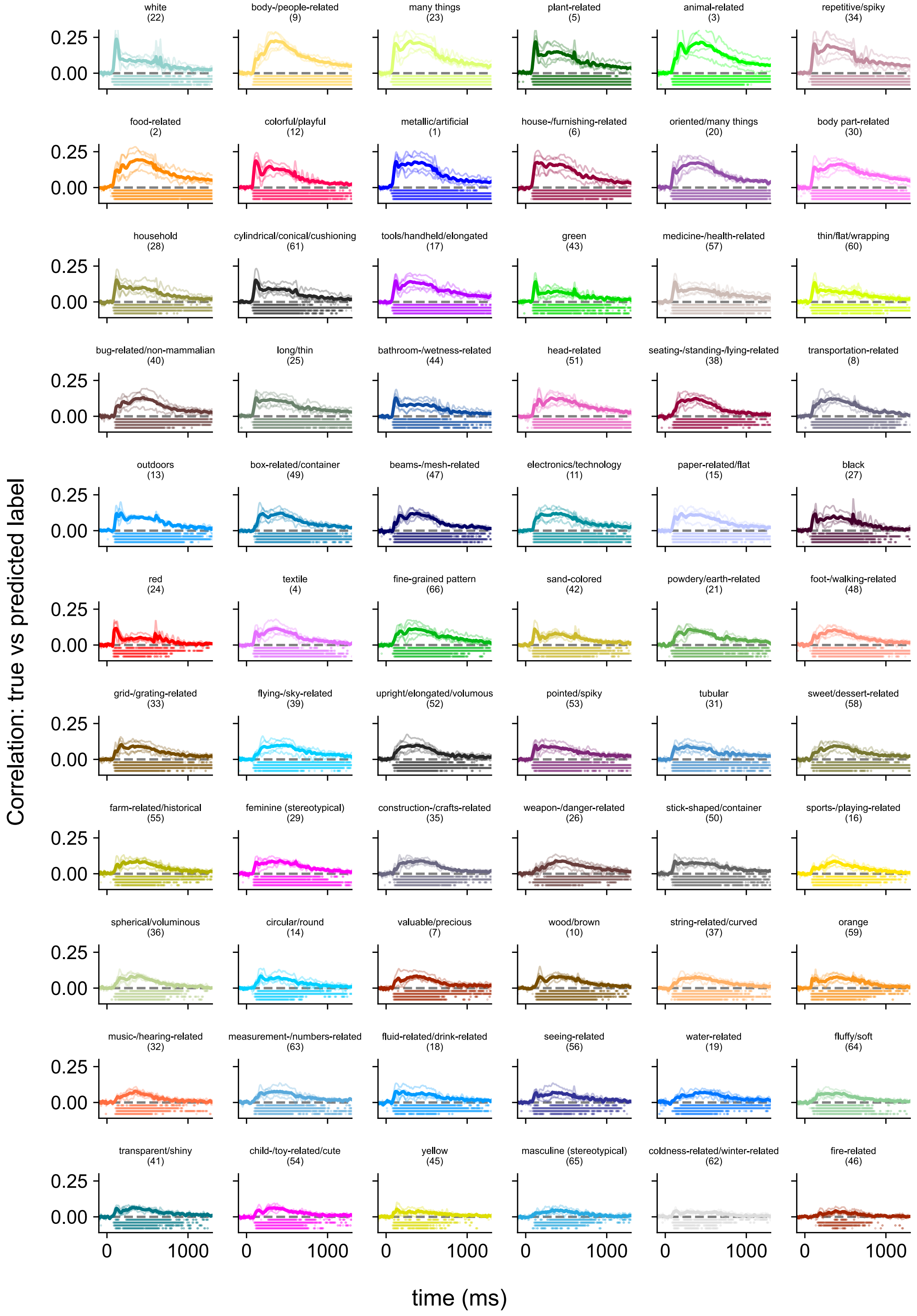

bioRxiv preprint doi: https://doi.org/10.1101/2023.09.08.556679; this version posted February 28, 2025. The copyright holder for this preprint (which was not certified by peer review) is the author/funder. This article is a US Government work. It is not subject to copyright under 17 USC 105 and is also made available for use under a CC0 license.

Figure S1. Dimension time courses for within-participant regression model. Each panel shows the correlation between the predicted and true weights for each dimension over time. The time courses are sorted by peak amplitude. The thin lines show the correlations for each participant. The thick lines show the average across participants. Dots below the timeseries show significant timepoints at $p < 0.01$ based on subject-level null distributions derived from permutations (see Methods).

bioRxiv preprint doi: https://doi.org/10.1101/2023.09.08.556679; this version posted February 28, 2025. The copyright holder for this preprint (which was not certified by peer review) is the author/funder. This article is a US Government work. It is not subject to copyright under 17 USC 105 and is also made available for use under a CC0 license.

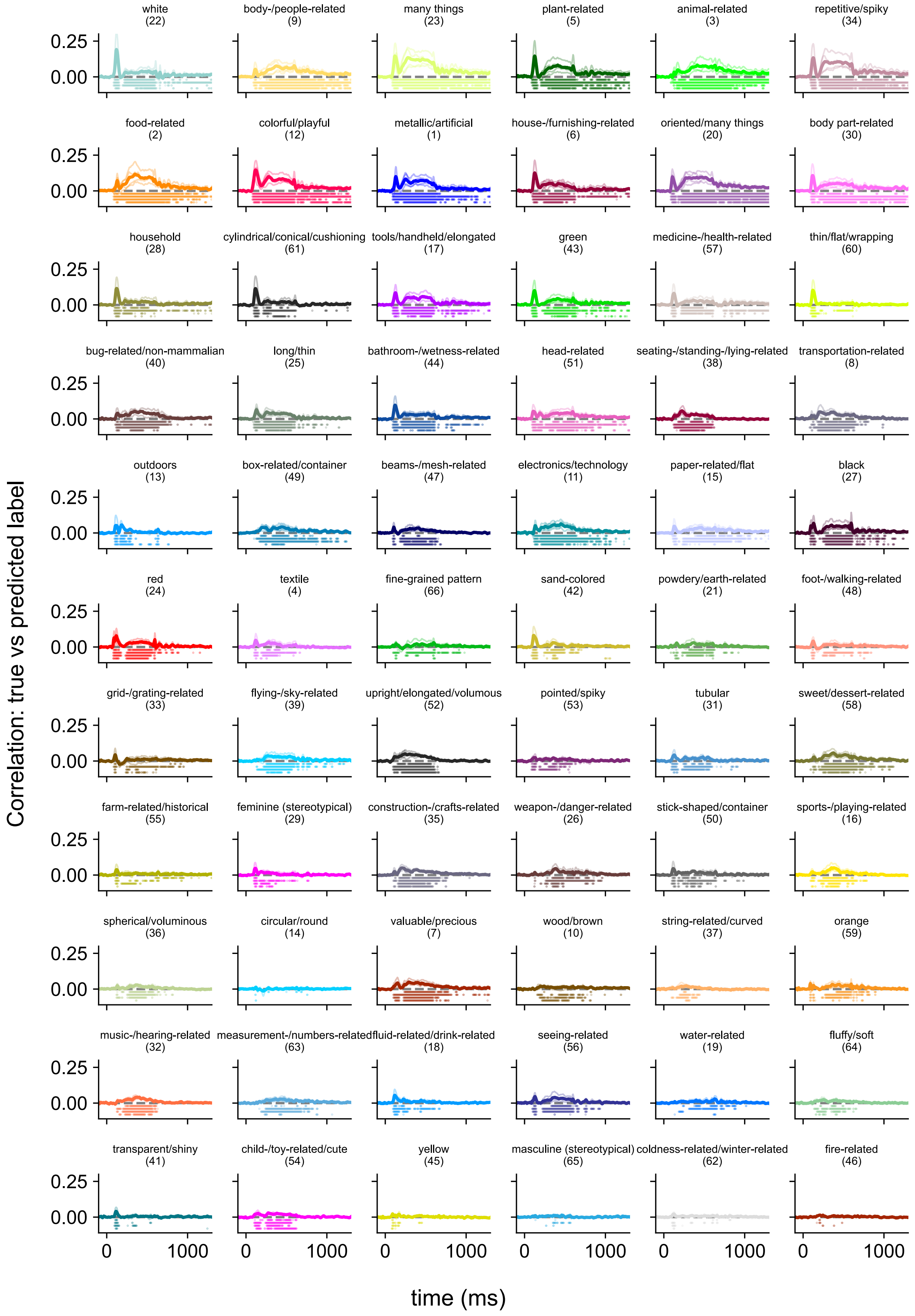

bioRxiv preprint doi: https://doi.org/10.1101/2023.09.08.556679; this version posted February 28, 2025. The copyright holder for this preprint (which was not certified by peer review) is the author/funder. This article is a US Government work. It is not subject to copyright under 17 USC 105 and is also made available for use under a CC0 license.

Figure S2. Dimension time courses for across-participant regression model. Each panel shows the correlation between the predicted and true weights for one of the 66 dimensions over time when the model is trained and tested on data from different participants. The thin lines show the correlations for each participant. The thick lines show the average across participants. Dots below the timeseries show significant timepoints at $p < 0.01$ based on subject-level null distributions derived from permutations (see Methods).

bioRxiv preprint doi: https://doi.org/10.1101/2023.09.08.556679; this version posted February 28, 2025. The copyright holder for this preprint (which was not certified by peer review) is the author/funder. This article is a US Government work. It is not subject to copyright under 17 USC 105 and is also made available for use under a CC0 license.

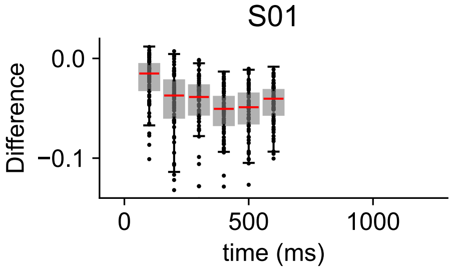

	100ms	200ms	300ms	400ms	500ms	600ms
100ms		.023*	.025*	.033*	.033*	.025*
200ms			.002	.011*	.010	.002
300ms				.009*	.008*	.000
400ms					-.000	-.008*
500ms						-.008*
600ms						

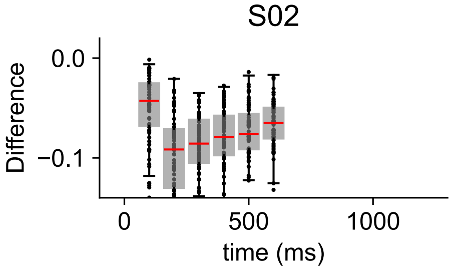

	100ms	200ms	300ms	400ms	500ms	600ms
100ms		.051*	.038*	.031*	.025*	.019*
200ms			-.013	-.020*	-.026*	-.032*
300ms				-.007	-.013*	-.019*
400ms					-.006*	-.012*
500ms						-.006
600ms						

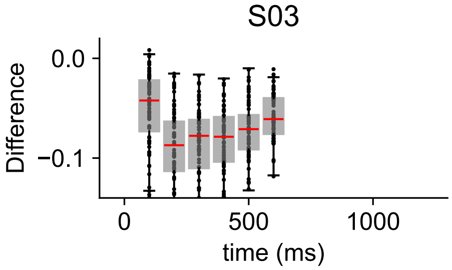

	100ms	200ms	300ms	400ms	500ms	600ms
100ms		.040*	.036*	.033*	.023*	.009
200ms			-.004	-.007	-.017*	-.031*
300ms				-.003	-.013*	-.027*
400ms					-.009*	-.024*
500ms						-.014*
600ms						

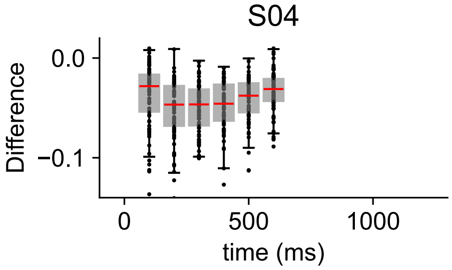

	100ms	200ms	300ms	400ms	500ms	600ms
100ms		.015*	.015*	.011	.006	-.002
200ms			-.001	-.004	-.010	-.018*
300ms				-.003	-.009*	-.017*
400ms					-.006*	-.014*
500ms						-.008*
600ms						

Figure S3. Differences in within- and across-participant models. Each row shows the data from one participant. The left panels show the difference between the within and the across participant model across the 66 dimensions at 6 different timepoints of interest. The right table shows the differences between each pairwise comparison of the timepoints of interest with green boxes (and stars) highlighting whether the comparison is significantly different from zero (Bonferroni corrected, $p < 0.01$).
