# Supplementary material for: Dynamic representation of multidimensional object properties in the human brain
Source: bioRxiv. 2024 Jun 13:2023.09.08.556679. Preprint. [Version 3] doi: 10.1101/2023.09.08.556679 (PMC10515754; doi:10.1101/2023.09.08.556679)
Supplement: Supplementary file 1 [file NIHPP2023.09.08.556679V3-supplement-1.pdf]

bioRxiv preprint doi: https://doi.org/10.1101/2023.09.08.556679; this version posted June 13, 2024. The copyright holder for this preprint (which was not certified by peer review) is the author/funder. This article is a US Government work. It is not subject to copyright under 17 USC 105 and is also made available for use under a CC0 license.

Supplementary Materials

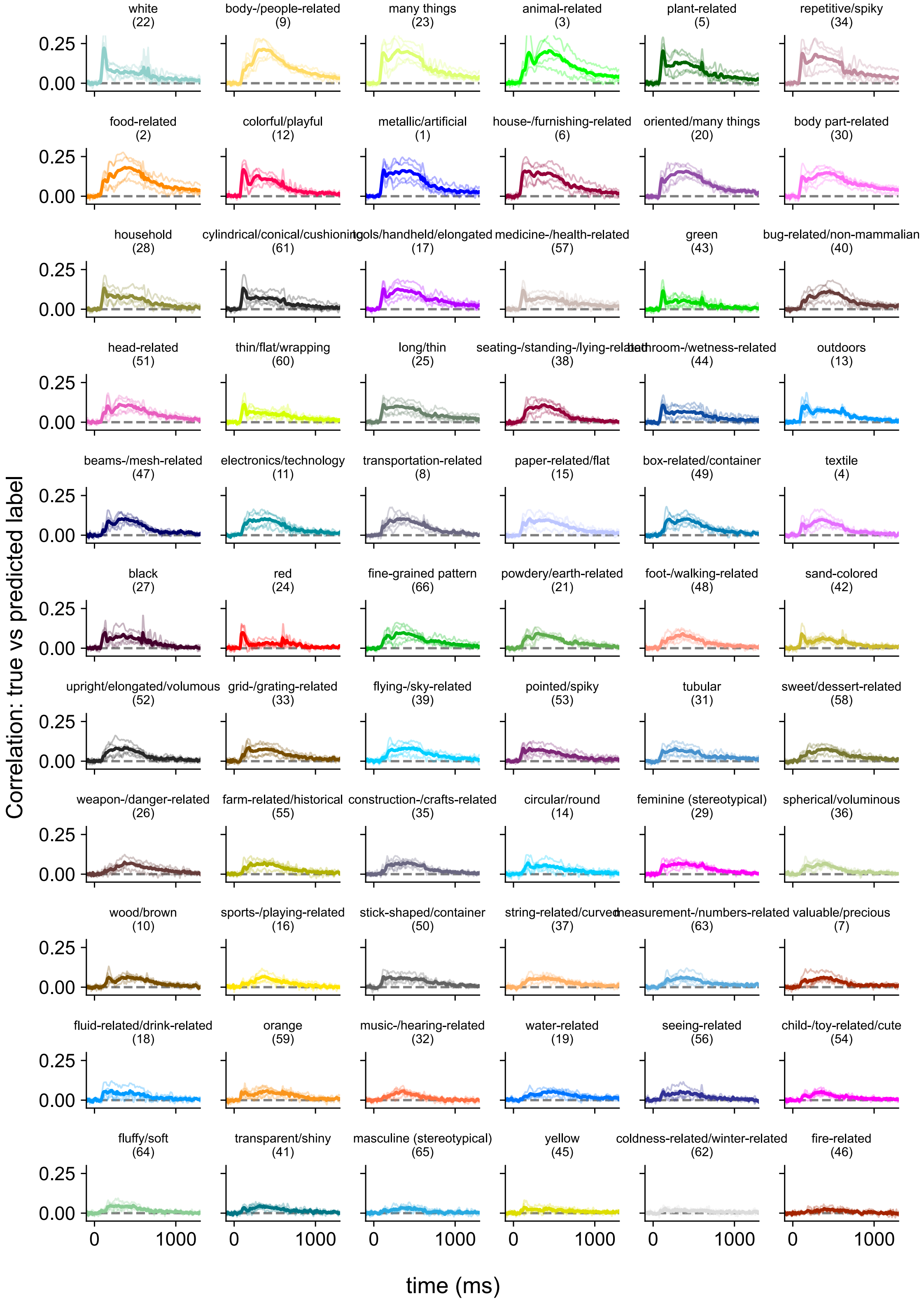

bioRxiv preprint doi: https://doi.org/10.1101/2023.09.08.556679; this version posted June 13, 2024. The copyright holder for this preprint (which was not certified by peer review) is the author/funder. This article is a US Government work. It is not subject to copyright under 17 USC 105 and is also made available for use under a CC0 license.

Figure S1. Dimension timecourses for within-participant regression model. Each panel shows the correlation between the predicted and true weights for each dimension over time. The timecourses are sorted by peak amplitude. The thin lines show the correlations for each participant. The thick lines show the average across participants.

bioRxiv preprint doi: https://doi.org/10.1101/2023.09.08.556679; this version posted June 13, 2024. The copyright holder for this preprint (which was not certified by peer review) is the author/funder. This article is a US Government work. It is not subject to copyright under 17 USC 105 and is also made available for use under a CC0 license.

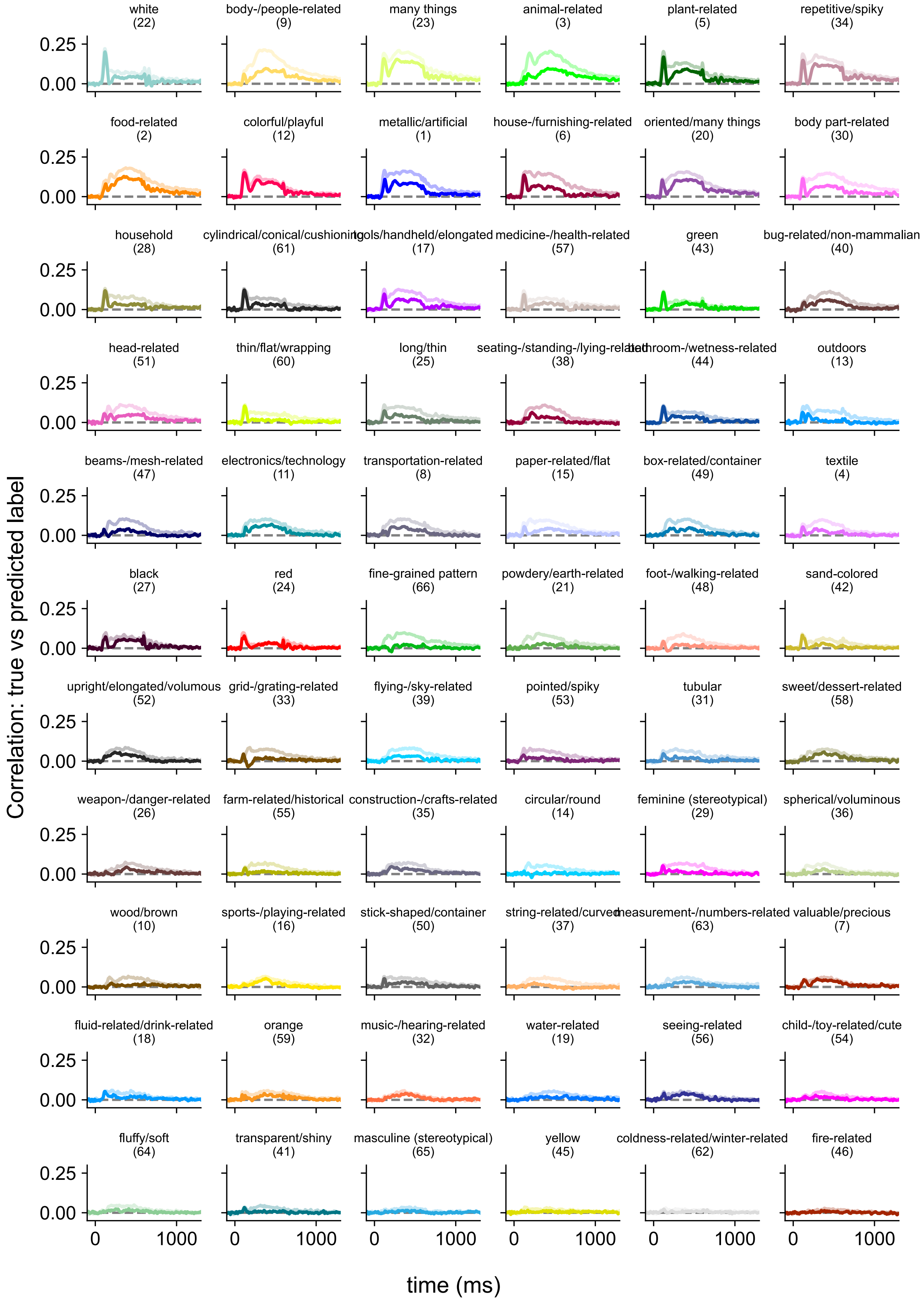

bioRxiv preprint doi: https://doi.org/10.1101/2023.09.08.556679; this version posted June 13, 2024. The copyright holder for this preprint (which was not certified by peer review) is the author/funder. This article is a US Government work. It is not subject to copyright under 17 USC 105 and is also made available for use under a CC0 license.

Figure S2. Dimension timecourses for across-participant regression model. Each panel shows the correlation between the predicted and true weights for one of the 66 dimensions over time. Saturated lines show the correlation timecourses for the across-participant models where the model is trained on data from three participants and tested on the data from a left-out participant. The transparent lines show the average correlation timecourses for the within-participant model as a reference. The timecourses are sorted by peak amplitude.
